# Modeling the Minimal Newborn's Intersubjective Mind: The Visuotopic-Somatotopic Alignment Hypothesis in the Superior Colliculus

**DOI:** 10.1371/journal.pone.0069474

**Published:** 2013-07-26

**Authors:** Alexandre Pitti, Yasuo Kuniyoshi, Mathias Quoy, Philippe Gaussier

**Affiliations:** 1 Department of Compter Sciences, ETIS Laboratory, UMR CNRS 8051, the University of Cergy-Pontoise, ENSEA, Cergy-Pontoise, France; 2 ISI Laboratory, Department of Mechano-Informatics, Graduate School of Information Science and Technology, University of Tokyo, Tokyo, Japan; McGill University, Canada

## Abstract

The question whether newborns possess inborn social skills is a long debate in developmental psychology. Fetal behavioral and anatomical observations show evidences for the control of eye movements and facial behaviors during the third trimester of pregnancy whereas specific sub-cortical areas, like the superior colliculus (SC) and the striatum appear to be functionally mature to support these behaviors. These observations suggest that the newborn is potentially mature for developing minimal social skills. In this manuscript, we propose that the mechanism of *sensory alignment* observed in SC is particularly important for enabling the social skills observed at birth such as facial preference and facial mimicry. In a computational simulation of the maturing superior colliculus connected to a simulated facial tissue of a fetus, we model how the incoming tactile information is used to direct visual attention toward faces. We suggest that the unisensory superficial visual layer (eye-centered) and the deep somatopic layer (face-centered) in SC are combined into an intermediate layer for visuo-tactile integration and that multimodal alignment in this third layer allows newborns to have a sensitivity to configuration of eyes and mouth. We show that the visual and tactile maps align through a Hebbian learning stage and and strengthen their synaptic links from each other into the intermediate layer. It results that the global network produces some emergent properties such as sensitivity toward the spatial configuration of face-like patterns and the detection of eyes and mouth movement.

## Introduction

A growing number of developmental studies raise that the newborn infant is prepared, evolutionarily and physiologically, to be born intersubjective [1]–[3]. Here, social cognition is thought to start at the very beginning of infant development [4]–[6], instead of at its achievement as Piaget proposed it [7]. The unmatured brain of the fetus is argued to be socially prepared to recognize human faces at birth, to make eye contact with others [8], to respond emotionally to biological motion and to imitate others with limited abilities. In this nature versus nurture debate, we propose to investigate what could be the minimal neural core responsible for the development of the neonate social brain. This work pursues some other investigations in which we modeled different aspects of fetal and infant development features with computer simulations [9]–[16].

Perhaps the most famous experiment in favor of neonate social engagement is the one conducted by Meltzoff, who showed that newborns are capable of imitating facial gestures off-the-shelf [17]. Although still under debate, neonate imitation suggests that the bonding of human newborns is either innate or acquired from an early imprinting of the body image. Whether these neural circuits are pre-wired or not, they necessarily influence the normal cognitive development of neonates to guide the spontaneous interactions in the physical world and in the social world. Meltzoff suggests that neonates interact with others *because* they are capable of goal-directed actions and *because* they recognize this genuine characteristic in others. He summarized this idea in his “like-me” theory [18] where he proposes that this mirroring mechanism between self and others could be based on a supra-modal representation of the body constructed from intra-uterine motor babbling experiences. Accordingly, this supramodal body image is supposed to identify organs and their configural relations that will serve him later for the cross-modal equivalence underlying imitation [19]. The successful replicating of neonatal imitation in monkeys by Ferrari argues further for the commonality of an early recognition mechanism in mammals' development, which may be based on “mouth mirror neurons” for facial and ingestive actions [20], [21]. Although the visual and motor cortices seem mature enough to support such system at birth, a subcortical scenario is more probable [22], [23], in which the subcortical units shape the cerebral cortex. This scenario may explain how a primitive body image could be accessible at an early age for sensorimotor coordination.

Consequently, the early functioning of the subcortical structures from the fetal stage appears very important for cortical development and therefore for the development of the social brain [6], [24]–[26]. Considering further the case of neonate face recognition, Johnson argues that the visual cortex is not mature enough before two months to support this function [27]. He proposes that a fast-track modulation model that includes the superior colliculus (SC), the pulvinar and the amygdala is at work in newborns for face detection, mood recognition and eye contact. He suggests also that this midbrain structure –dubbed as the CONSPEC model– includes an innate face-like visual pattern, nonplastic, that influences gradually the learning of a separate plastic cortical system, dubbed as the CONLERN model [28], [29]; a variant of this model has been given by [30], [31].

In so far, despite their appealling layouts, Meltzoff's and Johnson's models have been criticized for lacking evidence that *(i)* the visual motor pathway has feature detectors that would cause faces to be attractive [32], [33] and that *(ii)* motor outputs look actually the same from a third party perspective [34], which refers to the so-called correspondence problem [35], [36]. We propose nonetheless that a framework consistent with both viewpoints can be drawn based on the neural functioning of the SC. More precisely, the SC presents three relevant features that are potentially determinant for the building of a social brain [6].

First, SC supports unisensory processing in the visual, auditory and somatosensory domains accessible in a topographically-ordered representation to orient the animal to the source of sensory stimuli. Just as visual cues orient the eyes for tracking behaviors [37], somatosensory cues extend the motor repertoire for full-body representation, including the neck and the face [38]–[40]; the SC is coextensive with the pons, which is concerned with facial sensation, movement and vibro-acoustic sensation [41] and the face is represented in a magnified fashion with receptive fields [38]. Although the SC is a structure late to mature, the somatosensory modality is the first modality to be mapped in the third trimester of pregnancy [42], followed by vision with observations of occular saccades behaviors [43]. These aspects are important since some developmental studies attribute to SC a role in fetal learning using some form of vibro-acoustic stimulation to explain how the fetus is capable to sense and to learn through the body skin [44] and that SC is well-known as an important pathway for gaze shifting and saccade control [45], [46]. Second, the SC supports sensory alignment of each topographic layer. That is, the somatotopic organization (in the deeper layers) is not only topographic but also follows the design of the visual map (in the superficial layers) [38], [47]–[49]. Third, the intermediate layers exhibit ‘multisensory facilitation’ to converging inputs from different sensory modalities within the same region in space. As expressed by King, *“multisensory facilitation is likely to be extremely useful for aiding localization of biologically important events, such as potential predators and prey, (…) and to a number of behavioral phenomena”* [49]. Stein and colleagues underline also the importance of the multimodal alignment between visuotopic and the somatotopic organizations for seizing or manipulating a prey and for adjusting the body [47].

Collectively, these aligned colliculus layers suggest that the sensorimotor space of the animal is represented in ego-centered coordinates [39] as it has been proposed by Stein and Meredith [38] and others [50]; the SC is made up not of separate visual, auditory, and somatosensory maps, but rather of a single integrated multisensory map. Although comparative research in cats indicate that multimodal integration in SC is protracted during postnatal periods after considerable sensory experiences [51]–[53], multisensory integration is present at birth in the rhesus monkey [54] and has been suggested to play a role for neonatal orientation behaviors in humans. Moreover, while the difficulty to compare human development with other species has been acknowledged, *“some human infant studies suggest a developmental pattern wherein some low-level multisensory capabilities appear to be present at birth or emerge shortly thereafter”* [55].

**Figure 1:**
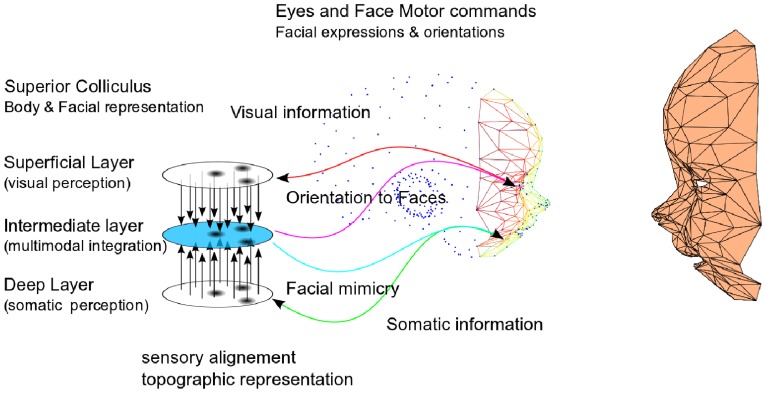
Proposal for a minimal network in SC for an inter-subjective mind. In comparison to normal stimuli, we propose that faces are particular patterns because the visual and somatic maps in the superior colliculus are perfectly aligned topologically in the intermediate layer. We suggest that the spatial distribution of the neurons in the somatotopic map is preserved in the intermediate map, which makes the multimodal neurons salient to visual patterns with a similar spatial configuration of eyes and mouth. We hypothesize that this feature potentially influence the social skills in neonates, for detecting faces and reproducing facial movements.

Considering these points about SC functionalities and developmental observations, we make the hypothesis that SC supports some neonatal social behaviors like facial preference and simple facial mimicry as a multimodal experience between the visual and somatosensory modalities, *not* just as a simple visual processing experience as it is commonly understood (see Fig. 1). We argue that, in comparison to standard visual stimuli, face-like visual patterns could correspond to unique types of stimuli as they overlap almost perfectly the same region in the visual topographic map and in the somatotopic topographic map. We propose therefore that the alignment of the external face-like stimuli in the SC visual map (some others' face) with the internal facial representation in the somatotopic map (one's own face) may accelerate and intensify multisensory binding between the visual and the somatosensory maps. Occular saccades to the correct stimulus may furtherly facilitate the fine tuning of the sensory alignment between the maps.

Moreover, in comparison with unimodal models of facial orientation, which support a phylogenetic ground of social development [31], [56], [57], this scenario would have the advantage to explain from a constructivist viewpoint why neonates may prefer to look at configurational patterns of eyes and mouth rather than other types of stimuli [25], [58]. Stated like this, the ego-centric and multimodal representation in the SC has many similarities with Meltzoff's suggestion of an inter- but not supra-modal representation of the body responsible for neonate imitation.

In this paper, we model the perinatal period starting from the maturation of unisensory layers to multisensory integration in the SC. This corresponds to the fetal maturation of the deep layers (somatosensory only) and of the superficial layer (vision only) at first, then to the post-natal visuo-somatosensory integration in the intermediate layers when the neonate perceives face-like patterns. Nonetheless, we make the note to the reader that we do not model the map formation in SC at the molecular level although there is some evidence that activity-independent mechanisms are used to establish topographic alignment between modalities such as the molecular gradient-matching mechanism studied in [59]. Instead, we focus at the epigenetic level, on the experience-driven formation of the neural maps during sensorimotor learning, in which we model the adaptation mechanisms in multisensory integration that occurs when there is a close spatial and temporal proximity between stimuli from different senses [60]–[64].

In computer simulations with realistic physiological properties of a fetus face, we simulate how somatosensory experiences resulting from distortions of the soft tissues (e.g., during the motion of the mouth or the contraction of the eyes' muscles) contribute to the construction of a facial representation. We use, to this end, an original implementation of feed-forward spiking neural networks to model the topological formation that may occur in neural tissues. Its learning mechanism is based on the rank order coding algorithm proposed by Thorpe and colleagues [65], [66], which transforms one input's amplitude into an ordered temporal code. We take advantage of this biologically-plausible mechanism to preserve the input's temporal structure on the one hand and to transpose it into its corresponding spatial topology on the other hand.

In comparison to other topological algorithms [67]–[71], the synaptic weights of each neuron inform about the vicinity to other neurons based on their rank order: that is, neurons with similar rank codes are spatially near. First, we study how the sensory inputs shape the sensory mapping and how multimodal integration occurs between the two maps within an intermediate layer that learns information from both. We propose that the registration of the somatosensory neural image aligned with the visual coordinates, as it could occur in the SC at birth, may give an easy solution to the correspondence problem, for instance, to recognize and to mimic the raw configuration of other people's facial expressions at birth. This scenario is in line with Boucenna and colleagues who showed how social referencing can emerge from simple sensorimotor systems [16], [72].

## Models

### Face Modeling

In order to simulate the somatosensory information on the skin, we use a physical simulation that verifies the average characteristics of a 7–9 months-old fetus' face. In our experiments, the whole face can move freely so that its motion can generate weak displacements at the skin surface and strong amplitude forces during contact.

**Figure 2:**
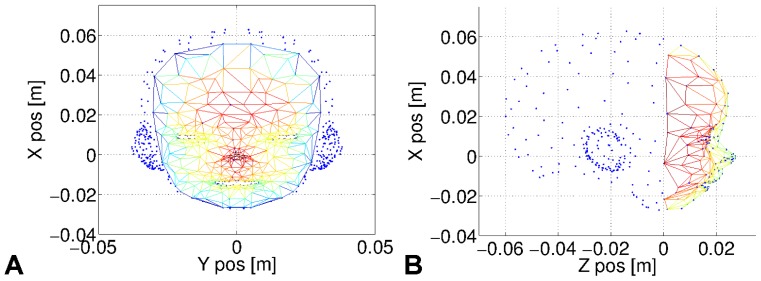
Face mesh of the fetus model. The distorsion of the facial tissue is simulated as a mass-spring network of 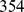 tactile points and 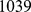 springs. Stress and displacement of the facial tissue are rendered by the actions of group muscles around the mouth and the eyes. In **A**, the front view of the face, the warm colors indicate the position of the segments in depth. The plot in **B**, the profile view, indicate the action limits of the face mesh in Z axis.

The face tissue is modeled as a mass-spring network and local stretches are calculated with the Hook's spring law (see below) representing the forces that a spring exerts on two points. The resulting forces on each node of the mesh simulate tactile receptors like the Meissner's corpuscles, which detect facial vibro-acoustic pressures and distortions during facial actions [73], see Fig. 2.
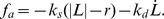


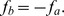


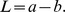


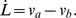



This formula represents the force applied to the particles 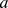 and 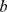; the distance between these particles, 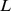; the rest length of the spring, 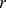; the spring constant or stiffness, 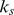; the damping constant, 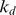; and the velocity of the particles, 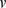. The damping term in the equation is needed in order to simulate the natural damping that would occur due to the forces of friction. This force, called viscous damping, is the friction force exerted on the mesh-network that is directly proportional and opposite to the velocity of the moving mass. In practice, the damping term lends stability to the action of the spring. The facial tissue is modeled with 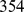 vertices and 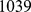 edges, and the mouth and the eyes apertures represent concave sets forming non-contiguous ensembles. The collision detection between two points or two springs is activated depending on the relative distance between the nodes and whether they are connected or not. On the one hand, for the case of contiguous points –that is, for the points connected with a spring– force collision is proportionnal to the local spring stiffness, to which no ad hoc force is added; this physical model corresponds to the behavior of the Meissner's corpuscles.

On the other hand, for the case of non-contiguous points –that is, unconnected points– virtual springs are added at the contact points to model the softness of the tissue material jonction and the stress in the radial direction; this physical model corresponds to the behavior of the Merkel cells, which are tactile receptors that detect pressure at localized points [74]. The radial force is added when the nodes' spatial location is below a certain minimal distance 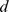 equals to 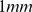.

For the sake of simplicity, we model the mouth motor activity and the eyes motor activity with virtual springs on the two lips of the mouth and on the two lids of the eyes. The contractions of these fictuous links control either the closure or the opening of the aperture of the mouth or of the eyes. In addition, we define as a prior choice that the two eyes move together (no eye blinking).

### Visual System Implementation

The eyes are the most controlled of the infant motor abilities at birth [46]. Although it is still unclear how and why visual system emerges during development, it has been argued that SC supports early visuomotor transformation [25].

Another proposal is that, before birth, traveling waves in the retina could serve as input to organize the formation of topological maps in the collicular visual system, furnishing preferential orientation and direction [75]. This process may be done even in the prenatal period because the eyes of the fetus can be seen to move in the womb from 18 weeks after conception, although the eyes stay closed until week 26 (6 months) [76], [77].

We model a rough eye receptive field to simulate this modality with a two dimensional matrix of 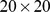 pixels (no log-polar transform), whose values are comprise between 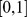 with no neighbouring information from each others. Morever, the eye position is considered fixed. We make the note that the topology respects the density distribution of the eye receptors in order to have more information on the fovea.

### Superior Colliculus Neural Model

Although there is little information about how non-visual information is translated into orienting motor input, numerous researches on fetal learning do report motor habituation to vibro-acoustic stimuli [44]. The exploration of the general movements in the womb are likely to generate intrinsic sensory stimuli pertinent for sensorimotor learning [41]. For instance, recent studies on the SC in the baby mole-rat indicate evidence for population coding strategies to accomplish orientation to somatosensory cues by a mammal, in a similar fashion to the treatment of visual cues and to eyes control in SC [40], [78], even at birth [46]. Other research further supports activity-dependent integration in the SC during map formation [60], [62], even though some molecular mechanisms are also at work [59].

Considering these points, we propose to model the experience-dependent formation of visuotopic and somatopic maps in the SC using a population coding strategy capable to preserve the input topology. We use for that the rank order coding algorithm proposed by Thorpe and colleagues [65], [79], which modulates the neuron's activation depending on the *ordinated* values of the input vector, *not* directly on the input values.

In comparison to Kohonen-like topological maps, this very fast biologically-inspired algorithm has the advantage to preserve the temporal or phasic details of the input structure during the learning, which can be exploited to organize rapidly the topology of the neural maps.

The conversion from an analog to a rank order code of the input vector is simply done by assigning to each input its ordinality 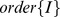 depending on its relative value compared to other inputs [66]. One neuron is associated to a specific rank code of the input units so that it is activated when this sequence occurs. A simple model of the activation function is to modulate its sensitivity based on the order in the input sequence 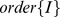 relative to its own ordinal sequence 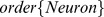, so that any other pattern of firing will produce a lower level of activation with the weakest response being produced when the inputs are in the opposite order. Its synaptic weights are learnt to describe this stage: 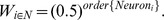 Its activation function is:
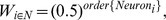


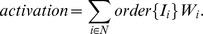



The most active neuron wins the competition and sees its weights updated according to a gradient descent rule:
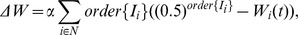
with 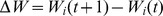 and 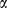 the learning step that we set to 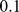.

By looking at the rank code in the weight vector, it is possible to measure and to compare the relative distance between the neurons, which respects the input topology.

During the learning process, we do not impose any lateral connectivity between the neurons. However, neurons with similar weights distribution may be considered neighbors and to belong to the same cluster. As we said earlier in this section, the process of map formation is done through the mechanism of activity-dependent neural growth [80]. However, we do not model the competition/stabilization processes at the molecular level as it has been described in [59]. Instead, we model here the neurogenesis and the neural spatialization with two complementary mechanisms. The first mechanism imposes to each neuron a maximum number of iterations above which its synaptic weights are no more plastic, we set 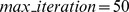. Besides, the second mechanism creates new neurons within the map initialized with plastic synapses when a neuron reaches its maximum allowed number of variation. This dual mechanism draws a developmental timeline by incrementing neurons within the maps where the most frequent stimuli patterns are represented with a greater number of neurons.

In our experimental simulations, the maps are initialized firstly with 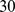 neurons and their maximum growth is fixed to 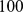 neurons. In accordance to the Model Section, the somatic map is linked to the 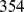 afferent somatic nodes and the vision map is linked to 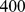 afferent retinal nodes. We make the note to the reader that each sensory map is unique which is different from the real anatomy of SC that is comprised of two hemispheres, each of which is mapped independently and organized such that central visual space along the azimuth axis is represented anteriorly and more peripheral space is posterior. We think nonetheless that our model is coherent and grasps the functional features of SC, like the sensory alignment. The experiments are presented therein-after in the next section.

## Results

### Development of Unisensory Maps

**Figure 3:**
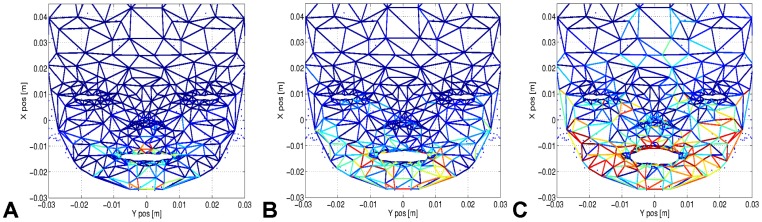
Strain/stress evolution of the facial tissue during the opening and the closing of the mouth. The figures highlight the propagation of the strain/stress lines on the facial tissue around the mouth during its opening. The color intensity indicates the variation on each edge of the relative stress, which is propagated from neighbouring points to neighbouring points. The tension lines permits to draw the functional connectivity of each region on the facial tissue.

**Figure 4:**
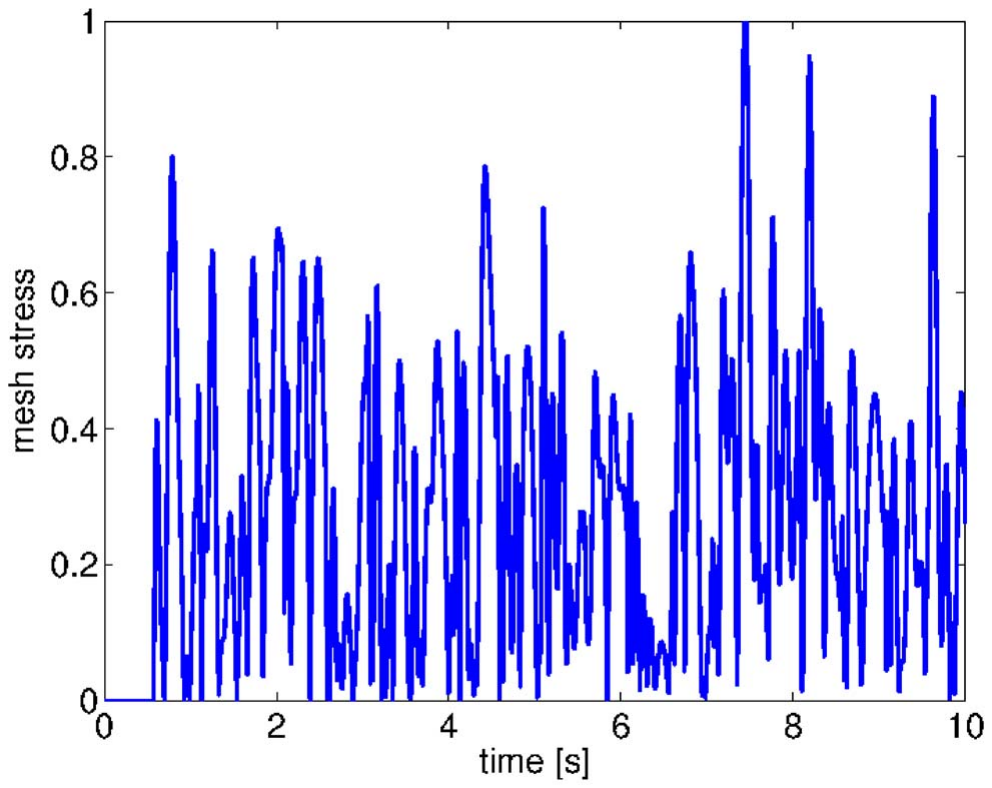
Stress intensity profile observed in one node. We can observe the very dynamic stress intensity level during facial movements on one node, normalized between 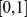. Its complex activity is due to the intermingled topology of the mesh network on which it resides. Some features from the spatial topology of the whole mesh can be extracted however from its temporal structure.

Our experiments with our fetus face simulation were done as follows. We make the muscles from the eyelids and from the mouth to move at random periods of time, alternating rapid and slow periods of contraction and relaxation. The face model simulates the tension lines, which propagate across the whole facial tissue, producing characteristic strain patterns mostly localized around the organ contours, see Fig. 3. Here, the stress induced by the mouth's displacement is distributed to all the neighbouring regions. These graphs show how dynamic the patterns are due to the intermingled relations within the mesh network. For instance, the intensity profile in only one node during mouth motion displays complex dynamics difficult to apprehend, see Fig. 4 for the normalized activity between 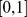. Thus, an important feature for a learning algorithm is to find the causal links and the topological structure from their temporal correlation patterns. The rank order coding algorithm satisfies these requirements because it allows to identify the amplitude relations among the tension nodes. The formation of the visual map follows a similar process. In order to mimic the visuo-spatial stimuli occuring when touching their face, we model the hand as a ball passing in front of the eye field and touching the skin at the same time (not shown). We make the note that occular movements are not modeled.

**Figure 5:**
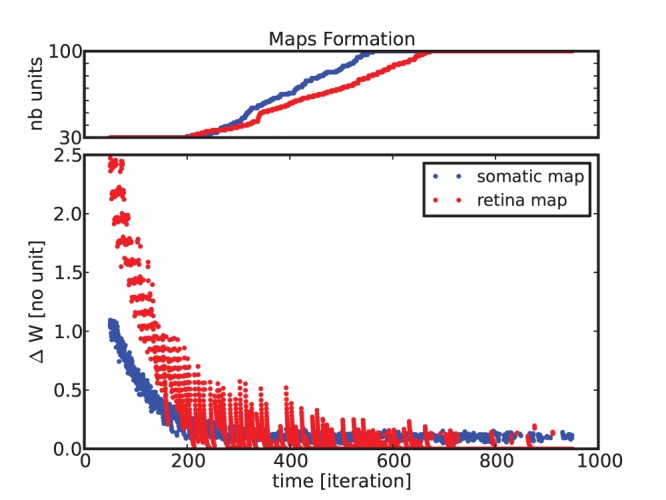
Evolution of the neural growth and synaptic plasticity during map formation. The plots describe the global variation of the synaptic weights and the number of units in each map, over time. The colors correspond respectively to the somatic map (in blue) and to the visual map (in red). Over time, the unisensory layers converge to stable neural populations through the mechanism of reinforcement learning (hebbian synaptic plasticity) as 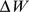 goes to zero and neurogenesis, as the maps reach their maximum number of units allowed; one hundred units. The density distribution of the neural populations depends on the sensory activity probability distribution.

During the learning process, the nodes from each map encode one specific temporal pattern and the most frequent patterns get over-represented with new nodes added. The developmental growth of the two maps is described in Fig. 5 with the evolution of the map size and of the weights variation parameter, 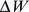, respectively top and bottom. While the convergence rate gradually stabilizes over time, new neurons get recruted which furnish some plasticity to the maps. After the transitory period, which corresponds to the learning stage, each neuron gets salient to specific receptive fields and 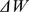 gradually diminishes.

**Figure 6:**
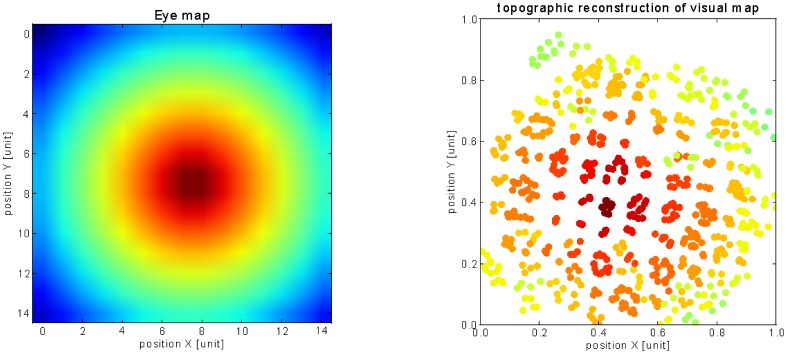
Visuotopic reconstruction using the Fruchterman-Reingold layout algorithm. This graphic layout (right) displays spatially in a 2D map the distance between neurons computed in the weights space on the principle of attraction/repulsion forces. The layout models grossely the molecular mechanisms of map formation. The graph shows that the visual neural network represents well the fovea-centered distribution of its visual input represented on the left with the same color code.

**Figure 7:**
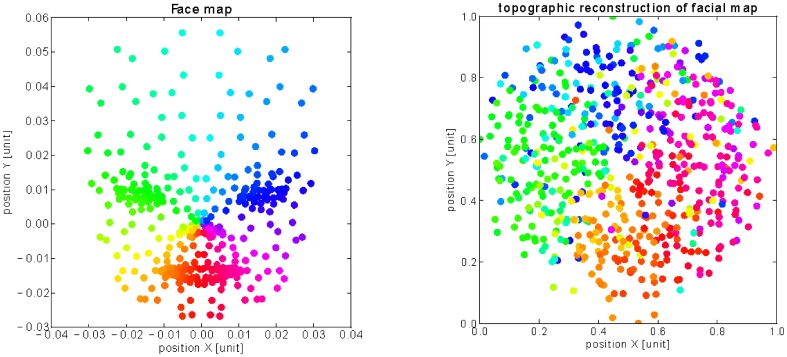
Somatopic reconstruction using the Fruchterman-Reingold layout algorithm. As in the previous figure, the Fruchterman-Reingold graphic layout (right) displays spatially in a 2D map the distance between neurons computed in the weights space for the tactile neurons, based on the principle of attracting and repelling forces. In accordance with the previous figure, the graph shows that the tactile neural network respects quite well the topology of the face (left) with the same color code for the neurons connected to their respective somatic area: the neural clusters respects the vertical and horizontal symmetries of the face with the orange-red-pink regions corresponding to the lower part of the face, the green-cyan-blue regions to the higher part of the face, the green and orange regions to left-side of the face and the blue-pink regions to the right-side of the face.

We reconstruct in Figures 6 and 7 the final configuration of the visuotopic and somatopic maps using the Fruchterman-Reingold (FR) layout algorithm [81], which is a force-directed graph based on the a measure distance between the nodes. Although very caricatural, the FR algorithm has been used for molecular placement simulations and can serve here to some extent to simulate the competition within the SC maps during ontogeny. We compute the euclidean distance between the weights distribution to evaluate the nodes' similarity and the attraction/repulsion forces between them. The color code used for plotting the visual neurons follows a uniform density distribution displayed in Fig. 6. Here, the units deploy in a retinotopic manner with more units encoding the center of the image than the periphery. Hence, the FR algorithm models well the logarithmic transformation found in the visual inputs.

Parallely, the topology of the face is well reconstructed by the somatic map as it preserves well the location of the Merkel cells, see Fig. 6. The neurons' position respects the neighbouring relation between the tactile cells and the characteristic regions like the mouth, the nose and the eyes: for instance, the neurons colored in green and blue are encoding the upper-part of the face, and are well separated from the neurons colored in pink, red and orange tags corresponding to the mouth region. Moreover, the map is also differentiated in the vertical plan, with the green/yellow regions for the left side of the face, and the blue/red regions for its right side.

### Multisensory Integration

The unisensory maps have learnt somatosensory and visual receptive fields in their respective frame of reference. However, these two layers are not in spatial register. According to Groh [45], the spatial registration between two neural maps occur when one receptive field (e.g., somatosensory) lands within the other (e.g., vision). Moreover, cells in true registry have to respond to the same visuo-tactile stimuli's spatial locations. Regarding how spatial registration is done in the SC, clinical studies and meta-analysis indicate that multimodal integration is done (1) in the intermediate layers, and (2) later in development after unimodal maturation [55].

**Figure 8:**
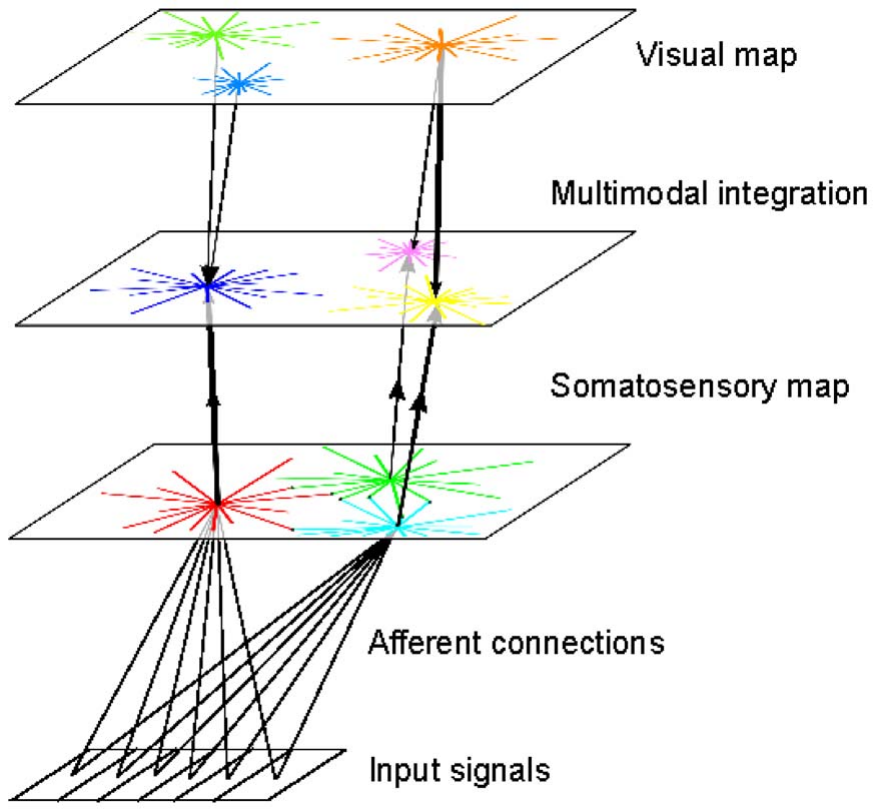
Multimodal integration schema in SC between vision and tactile information. Integration is done as follows, the visual signals in the superfical layer and the somatosensory signals in the deep layer converge to the intermediate multimodal map (no reentrance) in which bimodal neurons align pair-wise visuo-tactile associations. In certain cases, the synaptic links from different neurons in the unisensory maps converge to the same bimodal neurons whereas in other cases the synaptic links from the same neurons in the unisensory maps diverge to different bimodal neurons.

To simulate the transition that occurs in cognitive development, we introduce a third map that models this intermediate layer for the somatic and visual registration between the superficial and the deep-layers in SC; see Figs. 1 and 8. We want to obtain through learning a relative spatial bijection or one-to-one correspondence between the neurons from the visual map and those from the somatopic map. Its neurons receive synaptic inputs from the two unimodal maps and are defined with the rank-order coding algorithm as for the previous maps. Furthermore, this new map follows a similar maturational process with at the beginning 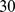 neurons initialized with a uniform distribution, the map containing at the end one hundred neurons.

**Figure 9:**
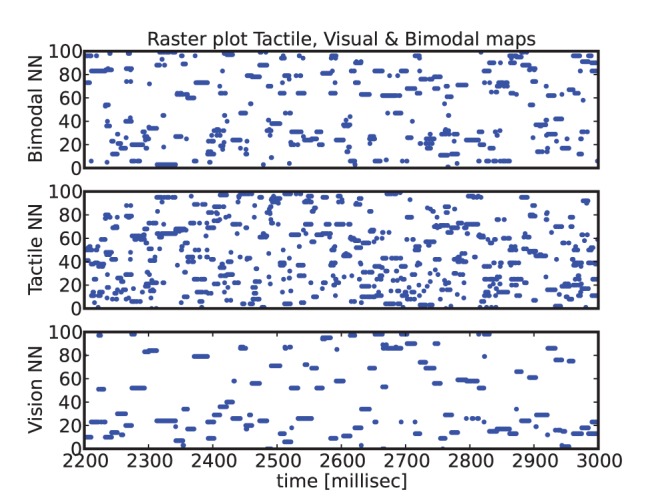
Raster plots from the visual, the tactile and the bimodal maps, during visuo-tactual stimulation when the hand skims over the face. The activity of the visual, tactile and bimodal maps is drawn respectively at the bottom, the middle and at the top frame. At a given time, the spikes contingency across the neurons in the three different maps creates the conditions for reinforcing their synaptic links from the neurons of the unisensory maps to the neurons of the bimodal map. The difference of spiking rates between the maps show that there is not a bijective connection between the neurons and that some bimodal neurons may associate groups of visual neurons to groups of tactile neurons.

**Figure 10:**
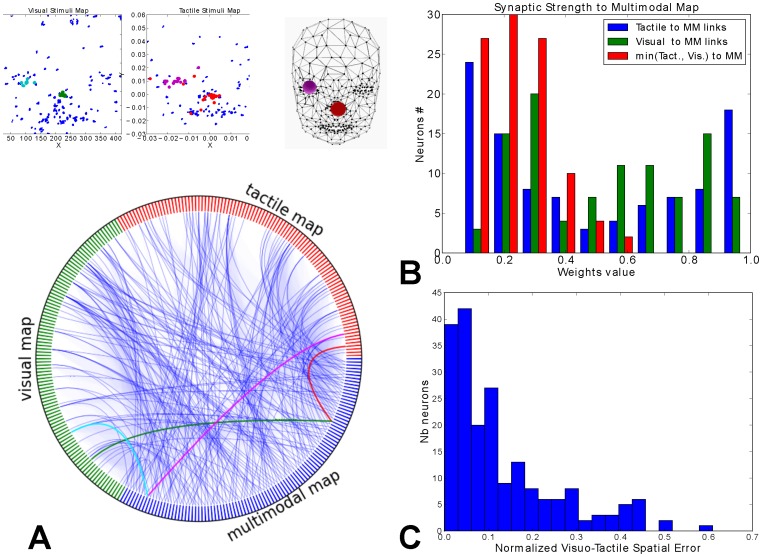
Networks analysis of visuo-tactile integration and connectivity. **A** Connectivity circle linking the visual and tactile maps (resp. green and red) to the bimodal map (blue). The graph describes the dense connectivity of synaptic links starting from the visual and tactile maps and converging to the multimodal map. The colored links correspond to localized visuo-tactile stimuli on the nose (green/red links) and on the right eye (cyan/magenta links), see the patterns on the upper figure. The links show the correct spatial correspondance between the neurons of the two maps. **B** Weights density distribution from the visual and tactile maps to the bimodal map relative to their strength. These histograms show that the neurons from both modalities have only few strong connections from each others. This suggest a bijection between the neurons of each map. **C** Normalized distance error between linked visual and tactile neurons. When looking at the pairwise neurons of the two maps (red histogram in **B** for weights 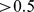), the spatial distortion between the neurons from the two maps is weak: vision neurons coding one location on the eyes receptive fields are strongly linked to the tactile neurons coding the same region on the face.

We present in Fig. 9 the raster plots for the three maps during tactual-visual stimulation when the hand skims over the face, in our case the hand is replaced by a ball moving over the face. One can observe that the spiking rates between the vision map and the tactile map are different, which shows that there is not a one-to-one relationship between the two maps and that the multimodal map has to combine partially their respective topology. The bimodal neurons learn over time the contingent visual and somatosensory activity and we hypothesize that they associate the common spatial locations between a eye-centered reference frame and the face-centered reference frame. To study this situation, we plot a connectivity diagram in Fig. 10 **A** constructed from the learnt synaptic weights between the three maps. For clarity purpose, the connectivity diagram is created from the most robust visual and tactile links. We observe from this graph some *hub-like* nodes in the bimodal map (the blue segment), which correspond to converging neurons from the two unimodal maps. Here, the intermediate neurons binds the two modalities. As an example, we color four links from the visual and tactile maps (resp. cyan, green and magenta, red segments) converging to two neurons from the bimodal map. We transcribe the associated visual and tactile patterns location at the top figures with the same color code. In these figures, on the left, the green dots in the visual map (resp. cyan and blue) indicate where the neurons trigger in visual coordinates and on the right, the red dots in the tactile map (resp. magenta and blue) indicate where the neurons trigger in tactile coordinates. Thus, the congruent spatial locations are mostly in registration from each others, and the bimodal map matches up with the two topologies.

In **B**, we reproduce the histogram distribution of the inter-modal connection weights taken from the tactile and visual maps to the bimodal map. The weights are uniformly distributed for the two modalities in blue and green with in average an equal number of weak connections (low values) and of strong connections (high values). However, for the neurons having necessarily strong links from both modalities (the red histogram), their number dramatically diminishes. For these neurons, only 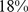 of the neurons population (i.e., eighteen neurons) have their synaptic weights above 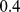 from the two unimodal populations. For neurons having their synaptic weights above 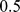, their number decreases to 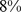 of the neurons population (i.e., eight neurons). Although the global nework is not fully recurrent, the probability distribution describes a log-curve distribution very similar to small-world and to complex networks [82]. Complex networks are well-known structures for efficient information processing, locally within the sub-parts and globally over the whole system [83].

**Figure 11:**
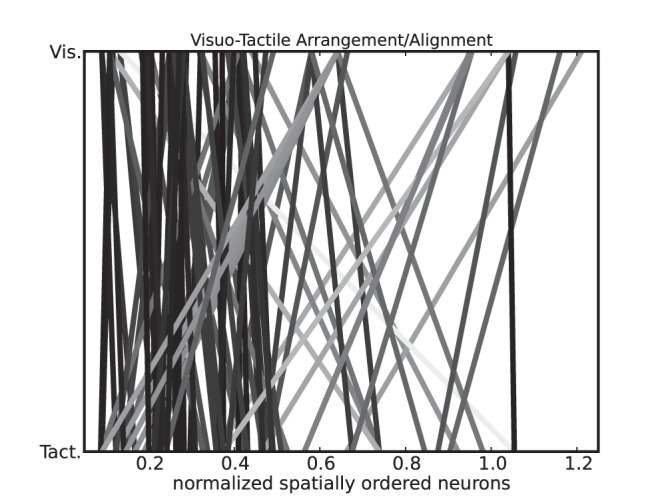
Neural arrangement and synaptic alignment. Spatial topology of the neurons in the visual and tactile maps, with their respective pairwise connections to the bimodal neurons, the darker the link, the more aligned are the neurons. In accordance with the results found in Fig. 9, the spatial error between the neurons of each map is weak, which is seen in the alignment of synapses that are mostly parallel; e.g., the dark links. At reverse, the few spatial errors present big spatial distortion (light grey).

The histogram in **C** draws a similar probability distribution for the spatial congruence between the visual mapping and the tactile mapping. This histogram displays the spatial error between the associated receptive fields taken from their respective barycentre (e.g., Fig. 10) and normalized between 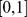. It shows that the unimodal receptive fields linked by the intermediate neurons overlap mostly their spatial location with 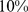 error only. Besides, the spatial distance decreases drastically above this value. As a result, most of the neurons from the two maps (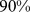) are in spatial registry. Figure 11 plots the spatial alignment between the visual and the tactile neurons, resp. above and below, relative to their location on their respective map. The links between the neurons are mostly vertical and parallel and only few of them cross other spatial regions on the other map. In order to mark out the aligned links, we color in dark grey the links that have a small spatial displacement between the two maps: the darker the link, the more aligned are the neurons.

### Sensitivity to Configuration of Eyes and Mouth

In order to investigate the functional properties of the global network, we replicate the three dots experiment tested on the newborns by Mark Johnson [8], [25]. This test aims at demonstrating facial imitation and facial perception in newborns.

**Figure 12:**
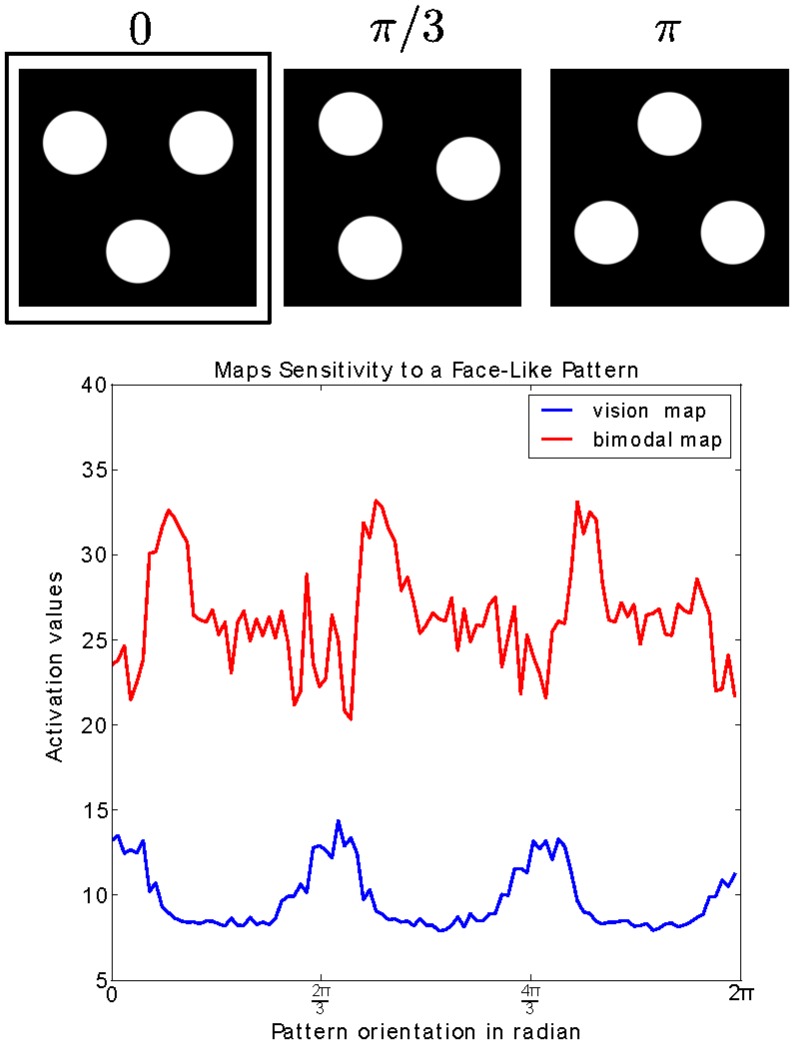
Sensitivity to face-like patterns for certain orientations. This plot presents the sensitivity of the neural network to face-like patterns, with an experimental setup similar to the three-dots test done in newborns [29]. When rotating the three dots pattern centered on the eye, the neural activity within the visual map and the bimodal map gets higher only to certain orientations, 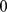 and 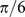, when the three dots align correctly to the caricatural eyes and mouth configurational topology.

We analyze the networks' activity response for different configurations of an iconified face-like pattern exemplified by three large dots corresponding to the two eyes and the mouth, see the framed figure in Fig. 12 on the top-left. For this, we rotate this pattern between 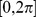 and collect the neural activation responses from the vision map (in blue) and from the intermediate map (in red). When the pattern is modulated by 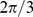 radians (120°), we can observe a strong response activation taken from the visual map as the face-like stimuli is well-aligned with the visual neurons, which have encoded this spatial distribution. Concerning the multimodal map, its neural response presents a similar activity pattern but two time stronger and shifted by 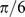 radians (30°). This slight difference in response between the two maps indicates that they share some common features in their respective receptive fields but do not completely overlap from each other. Although visual and somatosensory maps are not organized in the same manner due to the skin-based or retinotopic reference frames. As exemplified in Figure 11, the intermediate map recodes and aligns the two maps in a common space from the congruent visuo-tactile stimuli presented.

Furthermore, we can observe cross-modal enhancement as the activity in the multimodal map is higher than from its visual input. The face-like stimulation pattern boosts the neurons activity when they are presented in the correct orientation coinciding with the facial topology. Thus, activity in the intermediate layer is stronger despite it does not receive any information from the tactile map. That is, thanks to the sensory alignment between the two modalities, the intermediate layer is able to simulate the neural activity of the tactile map.

**Figure 13:**
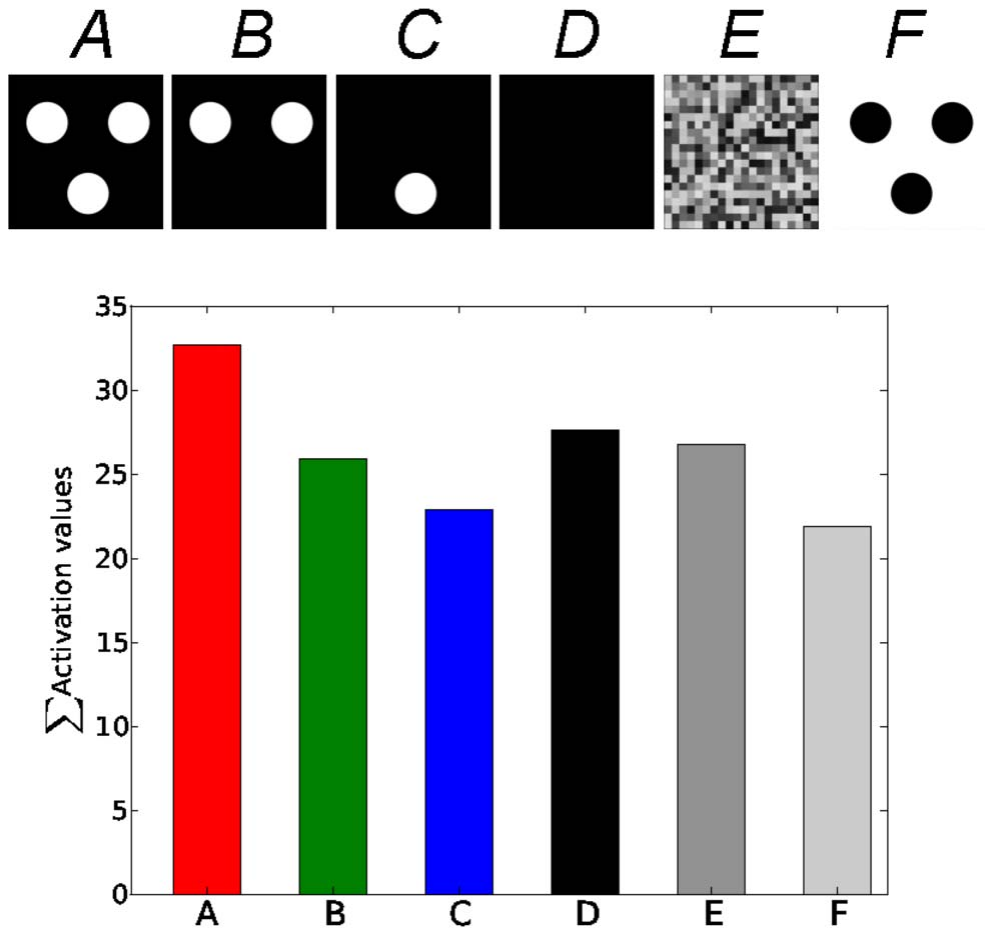
Performance Tests for different configurational patterns. We perform several experiments around the three dots test, the results on the sensitivity of the bimodal neurons are averaged on twenty experiments. In **A** the performance of the network on the black background and the three white dots, in **B** on the eyes only, in **C** on the mouth only, in **D** on a pitch black pattern, in **E** on a random pattern and in **F** on the reverse pattern. Bimodal neurons show a maximum intensity for the pattern **A**, where the three dots match the spatial location of the eyes and of the mouth. In comparison, its constitutive patterns presented separately to the network in **B** and in **C** generate a much lower activity, whereas The full back pattern in **D** and the random pattern in **E** reach an averaged activity level inside the network and the reversed pattern in **F**, its lowest level. This last performance is due to the contrast polarity sensitivity of the rank-order coding neurons, which is a characteristic comparable with the capacities of the visual system [65], but here the system learns light components against dark background but not dark components against light background as observed in infants [84].

In addition, we make five other experiments with different visual patterns in order to evaluate our system with respect to infants psychology tests. In Figure 13, we present the averaged activity level of the multimodal map over 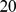 experiments, for the eyes and mouth configurational pattern with the white on black three dots **A**, the eyes only **B**, mouth only **C** and a black pattern, a random pattern and the black on white three dots pattern; resp. **D**, **E**, **F**. In this chart, the white on black three dots pattern in **A** is the most selective. In comparison to the eyes two dots pattern in **B** and to the one dot pattern in **C**, its level is much more higher than the sum of its constitutive patterns. Interestingly, a full black pattern, in **D**, or a random pattern, in **E**, get on average higher scores whereas the inverted three dots pattern in **F** gets the lowest level. Patterns **D** and **E** could correspond to the baseline of the map activity level, whereas pattern **F** show the contrast sensitivity of this type of neuron: rank-order coding neurons have been used to simulate the neurons in V1 and are found robust to noise and luminosity, but not to contrast polarity [65], [66], [79]. This point is particularly important because it may explain partly results on contrast sensitivity of neonates on face-like configuration [84], although neonates are more sensitive to black on white patterns rather than the reverse as in our model.

### Detection of Mouth and Eyes Movements

**Figure 14:**
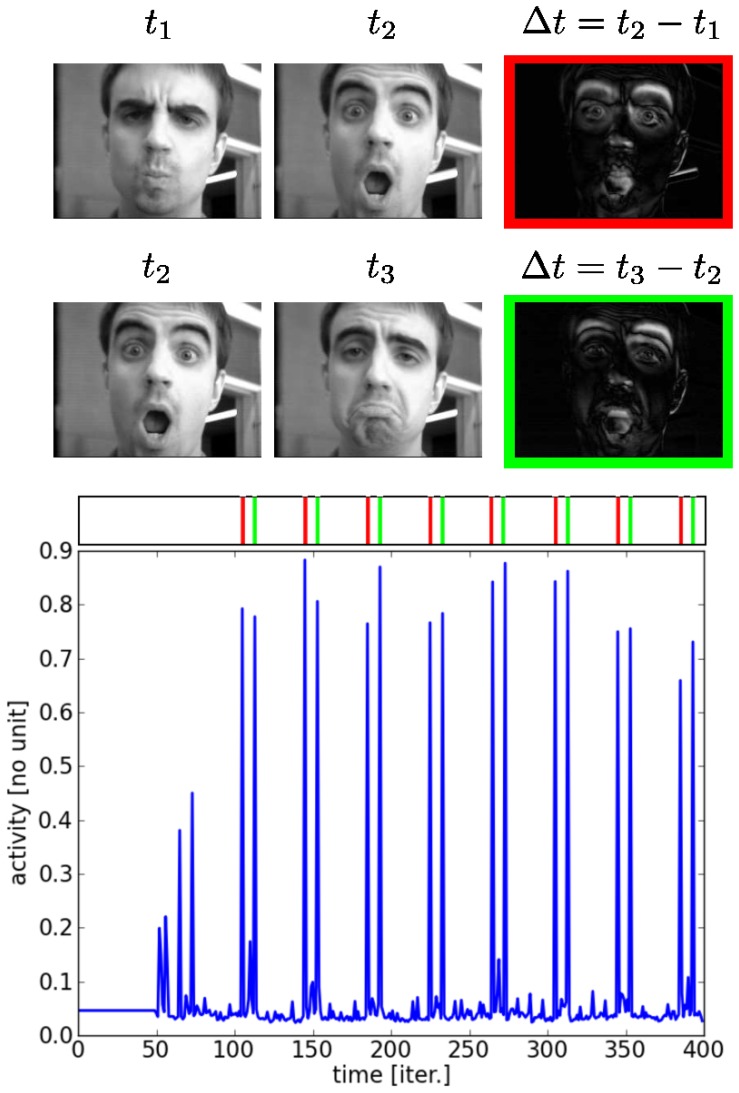
Neural activity taken from the intermediate visuo-tactile map during observation of a facial expression: surprise (red frame) and stare (green frame). We present a sequence of facial expressions from surprise to stare and vice-versa. The selected bimodal neuron taken from the intermediate map triggers to the characteristic visual configurational patterns of the face during rapid changes, which permits to detect the mouth and eyes movements. this behavior is due to the sensory alignment and of the high correlation with the tactile distribution of its own face. Note: the subject has given written informed consent to publication of his photograph.

Our next experiment studied the influence of facial expressions on the multimodal system. A sequence of facial expression images, which alternated stare and smile, is presented to the visual map at regular timing period. First, the images were pre-processed with a motion detection filter, which simply subtracts two consecutive images, see Fig. 14 on the top. As a result, the static regions between the two consecutive images are filtered (e.g., the background and the cheeks) whereas its dynamical parts (i.e., the eyelids, the eyes, the nose and the mouth) are strongly emphasized when a strong facial expression is established. In this situation, the salient regions match well the three dots icon in Fig. 12.

At the network level, not all the neurons are active but some are very receptive to certain facial expressions and to the dynamic activation of certain spatial regions. We display a neuron dynamics in Fig. 14 for different facial expressions presented at periodic time from staring to surprise, and then from surprise to staring.

Here, the visuo-tactile neuron in the intermediate map is visually highly receptive to the regions that characterize the face because of sensory alignment and that its distribution is correlated to the tactile distribution of its own face. Therefore, whenever a transition occurs in facial expression, the neuron fires. One can imagine then that if the intermediate cells feed-forward this activity to the corresponding facial motor activity, then imitation will occur.

## Discussion

We have introduced a developmental model of SC starting from the fetal stage in the context of social primitive behaviors. In comparison to normal stimuli, we propose that faces are particular patterns as the visual and somatic maps in SC are perfectly aligned topologically. We suggest that multimodal alignment may influence neonates for social skills, to recognize faces and to generate mimicry. The model consists of two unisensory layers, receiving the raw tactile information from the facial mechano-receptors simulated with a mass-spring mesh network and the raw visual information from the not-yet matured eyes. We make the note that the SC is comprised of two hemispheres and a unilateral SC lesion produces contralateral sensory (visual, somatosensory and auditory) deficits [85]. Although we could have modeled only one hemisphere and given to the system only half of the contralateral sensory information, we think our system would have learnt the same. The two circuits are initialized in a primitive stage starting with few neurons with randomized synaptic connections. We simulate the developmental aspects of the map formations during the third trimester of pregrancy through the mechanisms of activity-dependent neural growth [80] and synaptic plasticity. Over time, the two maps evolve into topographic networks and a third map is introduced, which corresponds to the intermediate layer in SC that aligns the visual and tactile sensory modalities from each other. The neurons are modeled with the rank-order coding algorithm proposed by Thorpe and colleagues [66], which defines a fast integrate-and-fire neuron model that learns the discrete phasic information of the input vector.

The major finding of our model is that minimal social features, like the sensitivy to configuration of eyes and mouth, can emerge from the multimodal integration operated between the topographic maps built from structured sensory information [86], [87]. A result in line with the plastic formation of the neural maps built from sensorimotor experiences [60]–[62]. We acknowledge however that this model does not account for the fine-tuned discrimination of different mouth actions and imitation of the same action. We believe that this can be done only to some extent due to the limitation of our experimental setup. In our predictions, however, we believe that a more accurate facial model which includes the gustative motor system can account to represent the somatopic map with more fine-tuned discrimination of mouth movements with throat-jaws and tongue motions (tongue protrusion) against jaw and cheeks actions (mouth opening). Moreover, our model of the visual system is rudimentary and does not show sensitivity in the three dots experiments of dark components against light background as observed in infants [84]. A more accurate model integrating the retina and V1 area may better fit this behavior.

Although it is not clear whether the human system possesses inborn predisposition for social stimuli, we think our model could provide a consistent computational framework on the inner mechanisms supporting that hypothesis. This model may explain also some psychological findings in newborns like the preference to face-like patterns, contrast sensitivity to facial patterns and the detection of mouth and eyes movements, which are the premise for facial mimicry. Furthermore, our model is also consistent with fetal behavioral and cranial anatomical observations showing on the one hand the control of eye movements and facial behaviors during the third trimester [88], and on the other hand the maturation of specific sub-cortical areas; e.g. the substantia nigra, the inferior-auditory and superior-visual colliculi, responsible for these behaviors [43].

Clinical studies found that newborns are sensitive to biological motion [89], to eye gaze [90] and to face-like patterns [28]. They demonstrate also low-level facial gestures imitation off-the-shelf [17], which is a result that is also found in newborn monkeys [20]. However, if the hypothesis of a minimal social brain is valid, which mechanisms contribute to it? Johnson and colleagues propose for instance that sub-cortical structures embed a coarse template of faces broadly tuned to detect low-level perceptual cues embedded in social stimuli [29]. They consider that a recognition mechanism based on configural topology is likely to be involved that can describe faces as a collection of general structural and configural properties. A different idea is the proposal of Boucenna and colleagues who suggest that the amygdala is strongly involved in the rapid learning of social references (e.g., smiles) [16], [72]. Since eyes and faces are highly salient due to their specific configurations and patterns, the learning of social skills is bootstrapped simply from low-level visuo-motor coordination. Besides, Meltzoff proposes that neonates possess an innate system named the Active Intermodal Matching (AIM mechanism) [19] that identifies organs and their configural relations. He furtherly suggests that this map is at the origin of a supramodal body image built from the visuo-motor matching behaviors, auditory-oral matching behaviors, and visual-tactile matching behaviors during the perinatal period [91].

How such body image can be built? and when? Takeshita and colleagues emphasize the importance of tactile sensation during brain maturation in the last trimester of pregnancy [92]. NIRS analysis on newborns during bimodal stimulation show that tactile stimuli activate in broader brain areas compared with other stimuli [93]. Retranscribed from [88], Kurjak and colleagues indicate that human fetuses begin to learn towards “their own body”, showing coordinated movements such as hands to mouth, sucking, grasping hand, tiptoes, knees (22 weeks), opening mouth before hand to mouth/sucking (24 weeks), and various patterns of facial expressions starting from 18 weeks (mouth opening, tongue/lip protrusion, smiling and yawning). Furthermore, supporting observations by Myowa-Yamakoshi and colleagues show evidences for fetal anticipatory mouth opening [94], whereas [43] shows continuity between fetal and neonatal neurobehavior with self-exploratory behaviors.

Although neonate imitation is only a marker that disappears after 2–3 months in human, we propose that the SC is at the root of this behavior for enabling automatic social interactions. This hypothesis has been also suggested by [95]–[97] who emphasized the central place that occupies the SC for fusioning the senses with respect to other brain regions not yet matured. Anatomical studies on collicular cells show that the eye neurons go forward to the deep layers without recurrent synaptic connections, which has to confer to SC a strong computational power due to alignment; e.g., the easy and rapid construction of a primitive body image. This primitive body image may correspond to the first-stage of Piaget's spatial and motor development landscape characterized by an egocentric representation and sensorimotor coordination before the apparition of a more complex spatial representation of the body in allocentric metric [7], [98], mapped into the cortex. The multimodal cells in SC, along with the other forebrain structures such as the hippocampus and the amygdala, may help the construction of such body schema in the parieto-motor cortices. For instance, we proposed in previous works the importance of hippocampal interactions with the parieto-motor cortices for spatial perception and the elaboration of a body image [15], [99]. There, other mechanisms than sensory alignment may be at play such as the gain-field modulatory effect found for coordinate transformation [100]–[103].
